# Child handwashing in an internally displaced persons camp in Northern Iraq: A qualitative multi-method exploration of motivational drivers and other handwashing determinants

**DOI:** 10.1371/journal.pone.0228482

**Published:** 2020-02-03

**Authors:** Julie Watson, Oliver Cumming, Robert Aunger, Claudio Deola, Rachel P. Chase, Robert Dreibelbis

**Affiliations:** 1 Department for Disease Control, London School of Hygiene and Tropical Medicine, London, England, United Kingdom; 2 Humanitarian Department, Save the Children, London, England, United Kingdom; 3 Independent researcher, Columbus, Ohio, United States of America; Bielefeld University, GERMANY

## Abstract

**Background:**

Children in humanitarian situations are particularly vulnerable to diseases such as diarrhoea. Handwashing with soap can greatly reduce transmission but handwashing rates are often low and traditional interventions ineffective. To aid future intervention design, this study aims to understand the determinants of child handwashing and the key motivational drivers of children’s behaviour within a specific humanitarian setting.

**Methods:**

In an internally displaced persons camp in Northern Iraq we conducted a series of 36 friendship-paired interviews with children aged 7–12 years, six semi-structured caregiver interviews, and three semi-structured hygiene promoter interviews. Perceived determinants of child handwashing were explored qualitatively, and motivational drivers were explored quantitatively with children in a rating exercise. Qualitative data were analysed thematically, using an inductive approach, and logistic regression analyses of motive rating data were performed to determine the predicted probabilities of motives being rated as important.

**Results:**

Access to soap and water was perceived to be high across all participant groups. Children, caregivers and hygiene promoters all perceive the determinants of child handwashing to be associated with familial role, environmental factors pertaining to location and quality of handwashing materials and facilities, and level of exposure to hygiene promotion, and children also attribute their handwashing to social norms. We find that children in this context are motived most by play and nurture.

**Conclusions:**

Provision of soap and water alone is not sufficient to encourage children to practice handwashing with soap in a humanitarian context. Our findings suggest that equal consideration should be given to the quality and location of handwashing materials and facilities and social norms could be leveraged to promote and enhance child handwashing. Motive-based interventions targeting play or nurture may be a promising approach and are likely most effective when used in conjunction, along with other motivational drivers such as affiliation and love.

## Introduction

Practising handwashing with soap at key occasions is a fundamental tool for the prevention of infectious disease. Handwashing with soap interrupts the transmission of infectious agents and can reduce the risk of diseases such as diarrhoea and acute respiratory infections by up to 23% and 21%, respectively [1, 2]. It has also been linked to the reduction of certain neglected tropical diseases with high disease burden in children, such as trachoma [3] and soil-transmitted helminths [4, 5], as well as lead to improvements in school attendance [6–8].

Despite the clear benefits of handwashing, rates are low; 81% of the global population fail to wash their hands with soap after defecating [1]. Though no official estimate of the handwashing rate among the global child population exists, age-segregating the studies comprising this overall estimate indicates that rates are even lower among children. Lack of handwashing is especially problematic in humanitarian emergencies where conditions such as overcrowding, unclean water and sanitation facilities, poor healthcare and environmental contamination leave people at high risk of disease [9, 10]. Diarrhoea can be responsible for up to 40% of all deaths in the immediate aftermath of an emergency [10]. Children in these contexts are particularly vulnerable, with diarrhoea and pneumonia being two of the leading causes of death in persons age fourteen and under [11].

The Sphere Handbook sets out minimum standards for hygiene promotion in humanitarian response [12] and most response agencies include handwashing promotion to both children and adults as part of their humanitarian response protocols. Multiple studies have documented that handwashing promotion approaches typically rely on a combination of communicating the health-related risks associated with poor hygiene and providing soap and water [13–16]. However, research from stable settings suggests that knowledge does not translate into practice and health is often not an effective motivator of behaviour change [13, 17, 18]. Though there is a paucity of research in this field from humanitarian settings, in refugee camps it has been shown that even when soap and water are present and handwashing is promoted via health-based messaging, rates of handwashing with soap are low [15].

To achieve success, behaviour change interventions must effectively identify and address the determinants (factors that influence behaviour) of the behaviour in question [19]. There have been a number of formative studies of the determinants of handwashing in stable settings [17, 20–22] however, these have largely focused on adult populations and to a lesser extent, children in schools [23–25]. There are as yet no published studies on the determinants of children’s handwashing behaviour in a humanitarian context.

Some behavioural theories place a strong emphasis on motives as determinants of behaviour and motivations have been explored in a number of the existing studies on handwashing determinants [17, 21]. According to the Evo-Eco theory [26], the theory at the centre of the Behaviour Centred Design (BCD) approach to designing and evaluating interventions [27], there are fifteen innate human motives that drive all human behaviour and have evolved to fulfil evolutionary important goals such as finding food or a long-term mate. Motivation-based handwashing interventions have shown promising results in stable settings, such as the SuperAmma campaign in India which used nurture, disgust, affiliation and status to motivate handwashing through animated film, skits and public pledging [28]. Until recently, however, motivation-based handwashing not been tested among children in humanitarian emergency settings.

Data presented here are part of a larger mixed-method study exploring the determinants of handwashing behaviours among children in an internally displaced persons (IDP) camp in Northern Iraq. In parallel to the study presented here, we tested the hypothesis that handwashing behaviour among children in this population could be determined by motivational drivers, as described in the Evo-Eco theory. We implemented a handwashing intervention organised around two specific motives—play and curiosity—and assessed its effect on children’s handwashing with soap in a proof-of-concept study. This study showed a large increase in rates of handwashing with soap among children [29].

Play and curiosity motives were selected *a priori* as primary drivers of children’s behaviour due to their intuitively assumed importance among this age group; however, it remains unknown whether these two motives are indeed the most important behavioural drivers among these children. An exercise of motive exploration could reveal which are the most important drivers of children’s behaviour and which could be most effectively targeted to produce robust behaviour change interventions in humanitarian settings.

In this multi-method study, we qualitatively explore the determinants of children’s handwashing behaviour in an IDP camp from the perspective of children, caregivers and hygiene promoters residing in the camp and we quantitively explore the most important motives driving children’s handwashing behaviour within this population.

## Methods

### Study site

The study took place in Sharia camp, an IDP camp located in the Dohuk Governorate of the Kurdistan Region of Iraq. This camp is managed by the Board of Relief and Humanitarian Affairs (BRHA)–a governmental body within the Dohuk government structure. At the time of this study, Save the Children were the organisation leading the water, sanitation, and hygiene (WASH) response in the Sharia camp. IDPs in the camp are exclusively from the Yezidi community, originating from the Sinjar region of Northern Iraq and most entered the camp in 2014 when the Islamic State of Iraq and the Levant (ISIL) entered Sinjar. Sharia camp has a population of approximately 17,000 people, over 37% of whom are children under the age of 12 [30]. The camp population is accommodated in tents with access to communal latrine blocks, shower units, and a consistent water supply at shared water points, though many families have purchased water tanks to store water in or near their tent. A previous study has shown that the rates of handwashing with soap among children after the five key moments (after using the toilet, before eating, before preparing food, after handling another child’s faeces, and before serving food to others) in this camp range from 13% to 32% [29].

### Participants and recruitment

72 children between the ages of seven and twelve participated in this study. They completed semi-structured interviews in 32 gender-segregated friendship pairs to encourage participation [31] (Table 1). In addition, we conducted semi-structured interviews with six female primary caregivers of children age 5–12, and semi-structured interviews with three camp-based hygiene promoters employed by Save the Children (one male, two female). All participants, including the hygiene promoters, were IDPs residing in the Sharia camp at the time of the study.

**Table 1:** Child friendship-paired interviews conducted with 72 IDP children.

Age group	Gender	Number of friendship-paired interviews
7–8	Female	6
7–8	Male	6
9–10	Female	6
9–10	Male	6
11–12	Female	6
11–12	Male	6

The Sharia camp is divided into five blocks (A—E). Primary caregivers and child participants were only selected from blocks not taking part in the concurrent proof-of-concept study (blocks A, C, and E) [32]. Two caregivers were recruited from each of the three eligible blocks. Each caregiver was recruited by randomly selecting a row of tents within one of the blocks (each block consisted of approximately 12–14 rows of tents) after which the lead author (JW) and local translator approached the first tent in this row. If the caregiver was not home or was ineligible, we moved on to the neighbouring tent, and so on until a caregiver was recruited from the row. All eligible caregivers we approached opted to participate. After written consent was obtained, the lead author and caregiver agreed a suitable time to conduct the interview. Interviews took place inside of the caregiver’s own tent for convenience and safety. The enrolled caregiver, lead author, and the local female translator, who provided translation for all interviews, were the only adults inside the tent during the interviews, although the young children of the household were sometimes also present.

Three hygiene promoters (two females, one male) were randomly selected from the group of six working in the Sharia camp. The lead author enrolled the hygiene promoters face-to-face and explained that she was in no way evaluating their abilities as hygiene promoters or working on behalf of their employer. All three hygiene promoters opted to participate, and interviews proceeded following written consent. Interviews took place in private in the Save the Children mobile office located in the Sharia camp.

Hygiene promoters assisted with recruitment of child participants, identifying households with at least one child age 7–12. For each friendship-paired interview, one child was recruited and then asked to nominate a friend of a similar age to join the interview. Assent and consent were sought respectively from both the children and their caregivers before proceeding with the interview. All nominated friends opted to participate, and all caregivers gave consent. Interviews took place in a private room within Save the Children’s child-friendly space in the camp in the presence of the lead author and translator. Children were guided to the room by Save the Children hygiene promoters who remained nearby on the premises to ensure safety.

Sample size was based on preliminary assumptions about the heterogeneity of the target population, housing and access to facilities, and logistical feasibility. We pre-defined six gender/age groups and completed a total of six interviews in each to reach assumed theoretical saturation [33]. As there were only six hygiene promoters working in the Sharia camp, theoretical saturation was expected within three interviews (i.e. 50% of this population).

### Data collection

All interviews were conducted between January 2018 and March 2018. Via the translator, all interviews were conducted by the lead author (a female academic researcher) who received training from colleagues and collaborating researchers. The lead author had no relationship with the participants prior to study commencement. Potential participants were informed about the nature of the study both informally and through a standardized document explaining that the study aim was to understand children’s handwashing behaviour in the camp and that data may ultimately help to shape future handwashing interventions. Interviews lasted between thirty minutes to one hour and were audio recorded, transcribed and translated verbatim. Following transcription and before analysis, each transcript was checked for accuracy against the original recordings by the lead author and translator.

Interview guides, specifically developed for each participant group by the authors and pilot tested prior to data collection, were used to guide the discussion on the topic of child handwashing in each interview. All interview guides can be found in S1 Appendix. Caregiver and hygiene promoter interview guides consisted of a series of semi-structured questions and child interview guides detailed a number of participatory tools which were used to elicit conversation around handwashing. An overview of these participatory tools is given in Table 2. A motive rating exercise was also undertaken with the child participants during the friendship-paired interviews and is detailed below.

**Table 2:** Participatory methods for friendship-paired interviews with IDP children.

Activity	Description	Purpose
Word associations	Handwashing-related words are called out and children describe what they associate this word with	To situate the conversation around handwashing and to understand the mental associations children have with handwashing and associated domains
Function of handwashing behaviour	Children list reasons for washing hands and then choose those most important to them, giving reasons	To understand the function handwashing serves from the perspective of the child
Routine scripting	Children recall their daily routine with the aid of picture cards and conversation is elicited around handwashing	To understand how handwashing features in daily routines and to identify barriers to practicing handwashing with soap
Pictorial vignettes of critical handwashing junctures	Pictorial vignettes depicting children in different handwashing scenarios are shown and children describe how they and others view the child, the reasons the child has washed/not washed their hands, and what may change the outcome	To explore social norms and barriers to handwashing
Ideal handwashing facility	Children describe their ideal handwashing facility and explain how it differs from their current facility	To elicit environmental barriers to practicing handwashing with soap
Perceived social norms	Children are given ten counters to represent children in the camp and are asked to estimate, giving reasons, how many have soap in their house, practice handwashing after using the toilet, etc.	To understand social norms around handwashing

To determine potentially important motivational drivers of children’s behaviour within this population, a motive sorting and rating exercise, was undertaken during the child friendship-paired interviews. Each child was presented with cartoon pictures depicting 14 of the 15 human motives from the Evo-Eco theory (the lust motive was deemed to be inappropriate for this age group and was excluded) [26]. The motive rating exercise was piloted prior to data collection using a number of different pictures and those pictures we felt were best understood were selected for use in the study. In addition, simple terms were consistently used to describe the pictures to the children in each interview (motive pictures and terms can be found in S2 Appendix). In the first phase of the motive rating exercise, each child was first asked to select the pictures that they felt were important to them with no restriction on the number they could choose. In the second phase, children were asked to select which motives were the most important to them. This staged process allowed all motives to be placed in one of three categories—not important, important, and very important.

### Data management and analysis

All interview transcripts were imported into QSR Nvivo 11 [34] to aid analysis. The lead author (JW) conducted a thematic analysis of all of the interview transcripts following the six stages described by Braun and Clarke: (i) becoming familiar with the data, (ii) generating initial codes, (iii) searching for themes, (iv) reviewing and naming themes, (v) defining themes, (vi) interpreting and reporting [35]. An inductive approach to coding was used to allow unexpected themes and concepts to emerge from the information provided by the participants. Codes identified features of the data considered to be of relevance to the research question. The coding structure can be found in S3 Appendix.

Statistical analysis of motive rating data generated from the child friendship-paired interviews was undertaken in Stata Version 14 [36]. The data violated the proportional odds assumption of ordered logistic regression so outcomes were dichotomised as ‘not important’ and ‘important or very important’ and a logistic regression analysis was undertaken for each motive to: (i) determine the predicted probability of the motive being rated as ‘important or very important’, adjusting for clustering within the child pairs (here, predicted probabilities translate to the predicted proportion of children who would rate a motive as ‘important or very important’), (ii) assess the association between motive rating and gender, controlling for age and adjusting for clustering within the child pairs, and (iii) assess the association between motive rating and age group, controlling for gender and adjusting for clustering within the child pairs.

Final predicted probabilities of motives being rated as ‘important or very important’ were then used to identify the smallest set of motives that could motivate the largest proportion of children. To do this we firstly identified the motive with the highest predicted probability of being rated as ‘important or very important’ (i.e. the motive that motivated the highest proportion of children). We then recalculated the predicted probabilities of motives being rated as ‘important or very important’ among only the children who had rated the previously identified motive as ‘not important’ and again we identified the motive with the highest predicted probability among this group (i.e. the motive that motivated the second highest proportion of children). These two motives were grouped, and the process continued until we had a set of motives with the potential to motivate nearly all children and where adding a further motive made little difference to proportion of children covered (i.e. motivated).

### Ethical considerations

The study was reviewed and approved by the London School of Hygiene and Tropical Medicine Ethics Review Committee (Ref: 14483) and the Hawler Medical University Ethics Review Committee in Erbil, Iraq (Ref: 1/16). The study was also approved by the Board of Relief and Humanitarian Affairs (Ref: 365) and the Directorate of Preventive Health Affairs in Dohuk Province (Ref: 7787). Written informed consent was obtained from all adult participants and caregivers of child participants and verbal assent was obtained from child participants.

## Results

### Perceived handwashing determinants

Four key themes emerged from the interviews describing the perceived determinants of child handwashing in the Sharia IDP camp. Three of these themes (familial roles, environmental barriers and hygiene promotion exposure) were common across respondent groups and so results were combined. The fourth theme (prescriptive social norms) was specific to the child friendship-paired interviews. All themes are detailed below.

#### 1. Familial roles

Across all three respondent groups, parental role was a key determinant of child handwashing. Lack of handwashing was seen as a reflection of poor parenting, and particularly attributed to a lack of care by mothers. Mothers were believed to carry the greatest responsibility because, as one hygiene promoter explained, *“she spends most of her time with them”*. Caregivers typically felt a lack of handwashing in the home was not due to a lack of resources but to family practices. One caregiver stated,*”*…*everything is available for the children and people in general*, *so I think it is something that depends on the family itself*.”

Caregivers and hygiene promoters believed that families with a large number of children find it harder to oversee their children’s handwashing. One hygiene promoter explained: *“I think the reason for this neglect may be the large number of children in the family*. *The father is not always at home and the mother remains busy with domestic work”*. This view was corroborated by caregivers:

“*Actually, as I see it, some of my neighbours have many children so it is hard to make them all wash their hands. Sometimes, when I see her children, I send them home and tell them to wash their hands*.”

Children also felt strongly about the familial role in child handwashing. The appearance of dirty hands was thought to be a reflection of poor parenting and a lack of handwashing in the home was attributed to parenting ability rather than resources. One child said *“Everything is available*, *but it depends on their parents*. *This boy's family is good and tells him to wash his hands*, *so he does but others do not wash their hands*. *There is nobody taking care of them*.*”*

In addition to the parental role, some children also felt that they played an important role in the handwashing of younger siblings. These children felt they acted as role models and should therefore demonstrate good handwashing so that younger siblings followed suit.

#### 2. Environmental barriers

Across respondent interviews, the common view was that availability of handwashing materials (soap, water and a handwashing station) was not an issue within the home. Most children (mentioned in 24 out of 36 friendship-paired interviews) and four caregivers reported having soap and water available in their home. Financial restraints were not a significant barrier to purchasing soap; only two caregivers and one child reported difficulties in affording soap in their households.

At the latrines however, respondents felt that many environmental barriers to child handwashing existed. One of these barriers was the availability of handwashing materials; water supply was reported to be intermittent at the handwashing stations within the latrine blocks and soap rarely available. Lack of lighting at the latrines was also a barrier to children practicing handwashing in the night. One child explained that, *“*…*some nights there is no electricity or water at the latrines*, *we are frightened to go to wash our hands*, *so it is hard for us to wash our hands*.*”* One caregiver also raised the lighting at the latrines as a concern and explained that she would have to accompany her children there because they felt frightened at night.

Other environmental barriers to children handwashing with soap were related to the conditions at the latrine blocks. In particular, dirtiness was perceived to be a significant environmental barrier to children practicing handwashing with soap there (although most reported that children would return home to wash their hands after using the latrines) and is also a psychological determinant, evoking the motive of disgust—a negative motivational driver for handwashing in this context. One child explained, *“we wash our hands at home because the latrines are dirty and also the water is dirty too”*. Another child said, *“if they (the latrines) are dirty I do not wash my hands in the latrines*, *I use latrines when they are clean”*. Caregivers also agreed that the dirtiness of the latrines was a problem and one gave the following account: *“Nobody can wash hands in the latrine because they are very dirty*. *Whoever enters will be dizzy because of the disgusting smell”*. In the instances where soap and water were available at the latrines, some children explained that they considered the soap to be too dirty to use because it was communal and kept in the unclean latrine block. They also complained that the water was too cold to use for handwashing, especially in the winter.

Most respondents attributed the dirtiness of the latrines to their communal nature and both children and caregivers believed that children’s handwashing would improve if households were provided with private latrine facilities. Private latrines were also desired because distance to the communal latrines presented a barrier to use of the handwashing stations there. One caregiver explained that, *“if they (latrine blocks) were closer*, *almost all of the children would be encouraged to wash their hands anytime they need to”*.

#### 3. Hygiene promotion exposure

Exposure to hygiene promotion among children in the camp was high; five of the six caregivers said that their children were exposed to hygiene messages and this was evident through the common knowledge of the seven steps of handwashing (mentioned in 18 of the 36 friendship-paired interviews) which are taught by hygiene promoters in schools, kindergartens and child-friendly spaces and the high awareness of disease. Both children and caregivers demonstrated knowledge of specific diseases that occurred in the camp, including cholera and mumps, and of disease transmission pathways. One child explained, *“we should wash our hands well because dirtiness goes under nails*, *so when we eat food*, *germs go into our body and we will be sick”*.

Avoidance of sickness and disease was stated as one of the most important reasons for practising handwashing in all of the child friendship-paired interviews. One child said, *“handwashing is so important for us to avoid cholera*. *We should wash our hands using soap*. *If we do not wash our hands*, *we will be sick”*. Children believed that knowledge of why and how to wash hands was an important determinant of handwashing and that hygiene promoters and parents should dispense this knowledge. Looking at a pictorial vignette depicting a child who did not wash her hands after using the toilet, one child stated that, *“if there is a CFS (child-friendly space) and they teach her the seven steps about handwashing she will know how to wash her hands”*. They felt that they also had a duty to impart knowledge to other children and another child explained, whilst looking at a pictorial vignette of a group of children with dirty hands: *“If we tell them all about handwashing*, *how handwashing is important and if you wash your hands you will be healthy*, *they will wash their hands”*.

Caregivers also believed that handwashing promotion was a key determinant of child handwashing. One caregiver said, *“if the hygiene promoters*, *CFS*, *and school tell them about handwashing they will wash their hands more because they like it and they will listen to you (hygiene promoters) more than us”*. Most agreed that their children enjoyed hearing messages from promoters and complained that household visits were decreasing, and NGOs were no longer doing enough to encourage children to wash their hands.

Hygiene promoters too felt that giving awareness was key to children’s handwashing. They believed that children’s handwashing in the camp had improved after Save the Children began operating in the camp and hygiene promoters believed that children who did not attend kindergartens and schools (and hence did not receive hygiene awareness there) were washing their hands less.

#### 4. Prescriptive social norms

Children expressed that a strong motivation for handwashing was to avoid social stigma. Normative importance of proper hygiene was a theme in 29 child friendship-paired interviews. One child said, *“if my hands smell good*, *people will not try to avoid me*, *and I will have lots of friends*.*”* Another explained, *“we want our hands to be clean and look to nice*, *so other children do not laugh at us”*. Talking about a child who does not wash his hands, another child said, *“if he is not clean people will always joke about him and his family”*. Children felt the reason that they or others would be avoided if they did not wash their hands was due to fear of catching disease. When shown a pictorial vignette of a child not washing their hands one child said, *“her friends will say to her ‘you are sick*, *so we cannot play with you or we will be sick like you’”*.

The idea of being stigmatised for not handwashing also extended to stigmatisation of the child’s family since handwashing was considered to be a reflection of the rest of the family’s cleanliness and social standing. When looking at a picture of a boy washing his hands, one child said, *“people will say he is clean*, *and he came from a clean family so maybe if you are clean it means your family is clean and asking you to be clean*.*”*

Children also washed their hands simply because their parents told them to and in order to gain their parents’ approval. One child said, *“our parents are proud of us when we wash our hands*, *they always encourage us to wash our hands*.*”*

All children in the friendship-paired interviews gave an estimate of the proportion of children in the camp that they believed would wash their hands with soap after using the toilet and the average estimate was 70%. Two of the hygiene promoters estimated the proportion of children practising handwashing with soap to be between 50%-60%. The other hygiene promoter did not give an estimate and neither did any of the caregivers.

### Motive analysis

Each child rated between two and eleven of the fourteen motives as ‘important’ or ‘very important’ (typically between two and five). Between zero and five motives were rated as ‘very important’ (typically one or two). Play had the highest probability of being rated as ‘important’ or ‘very important’ (56%), followed by nurture (54%), affiliation (47%) and comfort (46%). Predicted probabilities of each motive being rated as important or very important are shown in Table 3.

**Table 3:** Predicted probabilities of motive rating using logistic regression.

Motive	Predicted Probability of ‘important’ or ‘very important’ rating
Play	0.56
Nurture	0.54
Affiliation	0.47
Comfort	0.46
Hunger	0.40
Attract	0.38
Love	0.36
Create	0.36
Justice	0.28
Curiosity	0.31
Disgust	0.21
Fear	0.22
Hoard	0.21
Status	0.08

Analyses are all adjusted for clustering within the child pair.

N = 72 IDP children

Females had three times higher odds of rating the motive love as ‘important or very important’ compared to males, controlling for age (p<0.05; see Table 4). Controlling for gender, the odds of the motive love being rated as ‘important or very important’ were ten times higher for children in older age groups, 9–10 and 11–12, than for children age 7–8 (p<0.05). The oldest age group (ages 11–12) were at three times higher odds of rating the motive justice as ‘important or very important’ compared to the youngest age group (ages 7–8).

**Table 4:** Logistic regression analysis relating gender and age group to an ‘important’ or ‘very important’ motive rating.

Motive	Gender (Female vs. Male)		Age group 9–10 (vs. 7–8)		Age group 11–12 (vs. 7–8)	
	OR	P value	OR	P value	OR	P value
Play	1.25	0.62	1.13	0.84	0.99	0.99
Nurture	1.41	0.51	1.38	0.61	2.04	0.24
Affiliation	1.98	0.16	0.83	0.77	0.87	0.80
Comfort	1.75	0.23	0.81	0.69	0.42	0.15
Hunger	0.56	0.27	1.62	0.46	1.79	0.38
Attract	0.70	0.48	2.34	0.19	1.58	0.46
Love	2.99	0.01	10.89	<0.01	10.02	<0.01
Create	0.78	0.63	1.30	0.68	0.71	0.55
Justice	1.01	0.99	5.29	0.06	6.00	0.03
Curiosity	0.77	0.67	1.26	0.76	2.41	0.24
Disgust	2.42	0.14	0.10	0.05	1.42	0.56
Fear	1.98	0.29	1.48	0.66	3.35	0.07
Hoard	0.42	0.13	0.63	0.46	1.10	0.87
Status	0.88	0.88	0.41	0.31	1.00	1.00

Logistic regression controlling for age (in gender analysis) and controlling for gender (in age group analysis). Analyses are all adjusted for clustering within the child pair. N = 72 IDP children

A combination of the four motives: play, nurture, affiliation and love, were considered motivational by 96% of the children sampled (i.e. 96% of these children rated least one of these motives as important or very important) (Table 5).

**Table 5:** Motivation coverage across 72 IDP children.

Motive Combination	Predicted proportion of children motivated
Play only	56%
Play and Nurture	82%
Play and Nurture and Affiliation	91%
Play and Nurture and Affiliation and Love	96%

## Discussion

This is the first study of which we are aware to explore the determinants of child handwashing and the motivational drivers of child behaviour in a humanitarian emergency context. We found that children, caregivers and hygiene promoters in an IDP camp in Northern Iraq all perceived the determinants of child handwashing to be around familial role, environmental factors—including location and quality of handwashing materials and facilities, and level of exposure to hygiene promotion, and that children also perceive social norms to be an important determinant. We also found that children in this context are motived most by play and nurture.

Across all three interviewee groups, availability of soap and water at the household level was thought to be high and not a barrier to handwashing. This corroborates the high prevalence of handwashing stations, with soap and water present, observed in households during the baseline data collection activities of the concurrently implemented proof-of-concept study [29]. These handwashing stations were generally located next to the kitchen area, however tents in the Sharia camp are small and there was little distance between the handwashing station and any area within the household. In line with previous handwashing studies among adults in humanitarian settings [15, 37], our study suggests that providing a sufficient supply of soap and water during humanitarian emergencies, as there was in the Sharia camp, is not enough to achieve good handwashing practices among children.

We find that the quality of communal handwashing materials and facilities, and their location, are important determinants of child handwashing. These environmental determinants, particularly the dirtiness of materials and facilities (a key theme across the interviews), may be most relevant in humanitarian settings where latrines and their associated handwashing stations are often far from households, and difficult to maintain; however in stable settings, studies have also shown an association between washroom cleanliness and adult handwashing [38]. In the Sharia camp we found that dirtiness evoked the emotion of disgust, driving children to avoid the dirty area (the latrine blocks), and hence handwashing there. An abundant literature from stable settings supports disgust as a key motivator of handwashing [17, 21, 28, 39, 40] but our study highlights that when the source of disgust is the handwashing facility or it’s environment, as is likely in most humanitarian settings, this motive can also have the opposite behavioural effect. Furthermore, disgust of the environment and facilities may be a stronger behavioural driver than disgust of dirty hands. We note the discrepancy between this finding and the motive rating exercise—disgust was not rated as a particularly important motivational driver of behaviour. While we can’t say for certain, this may be in part because when thinking about behaviours, children have a tendency to think more of those with a positive association and because the disgust emotion stems from the automatic part of the brain, and when consciously considered with the executive brain, it is not recognised as a very important behavioural driver [21, 26].

To encourage children to practice handwashing with soap, we should ensure handwashing facilities are clean, have a consistent supply of water that is warm and soap that appears clean, are well lit, and are located close to latrines. Alternatively, providing private latrines in camps where households have their own handwashing stations of acceptable quality may also lead to improvements in post-latrine child handwashing practices. Though no previous studies have assessed the effect of latrine location on children’s handwashing in a humanitarian setting, having a handwashing station within 10 paces of the toilet was associated with an increase in adult handwashing in a stable context [20].

We find participants of this study hold a strong expectation that families, especially mothers, should take responsibility for children’s handwashing. This may have been accentuated by the camp setting; families live in close proximity within tents and extended family members often occupy neighbouring tents giving them ample opportunity to oversee the behaviour of others. Living in close proximity to others and sharing communal WASH facilities also likely strengthened the influence of social norms on children’s handwashing behaviour; children reported that they performed handwashing to appear clean in order to avoid social stigma of themselves and their families by their community and so that other children would play with them. Similarly, in a school environment in Bangladesh, the presence of another person, particularly another child, was found to increase children’s handwashing, and shame and stigma are also drivers of hygiene behaviour among adults [41]. These social norms may be a useful tool in future handwashing promotion interventions for children living in camp settings.

Children also felt that they could play an important role in the handwashing of their younger siblings, and they rated nurture (applied to themselves, not their parents) as one of the most important motives. This may be because children in the Sharia camp often take on caregiver roles; in the friendship-paired interviews, nine children reported a role in caring for younger siblings. The camp setting likely perpetuates this duty of care—children share a small living space with their siblings, spend more hours in the home than they did prior to displacement (due to less time in school), and some have lost a parent due to displacement-related causes. Thus far, nurture has only been used in motive-based behaviour change interventions for adult caregivers in stable settings [28, 42]. Our study suggests that, in humanitarian emergencies, the nurture motive could be useful to drive children’s handwashing behaviour. This may also be true in stable settings; Grover et al (2018) observed children in schools assisting younger children in handwashing and modelling handwashing techniques for other students [43].

Our study also adds support for the use of play in motive-based handwashing interventions for children in humanitarian emergencies. Children rated play as the most important motive in this study and this was one of two motivational drivers used in the concurrently-implemented proof-of-concept study [29]. It is noted that curiosity was also used as a motivational driver in the intervention study but was not rated as very important in our study. This may be because the intervention motivated children to wash their hands through play alone, or because curiosity was inaccurately conveyed in our pictures, a possibility given the notion of curiosity is more abstract than play. Not all children are highly motivated by the same drivers and, to have the greatest impact, motive-based interventions should consider using a set of motives. We find that in the Sharia camp an intervention targeting a set of four motives—play, nurture, affiliation and love—has the potential to motivate almost every child.

Perceived rates of handwashing with soap among children in the camp (50%-60%) were notably higher than actual rates measured in the concurrently-implemented proof-of-concept study (13%-32%) [29]. This disparity is likely attributed, in part, to the fact that perceived rates pertain to handwashing after toilet use, whereas actual rates pertain to handwashing at five key occasions. Nonetheless, perceived rates were high and may reflect the norms around social stigma discussed above—children believe that handwashing is occurring in private at the household more frequently than it is. Though we didn’t measure the actual handwashing rates of each child in the paired interviews, studies have found a positive correlation between perceived peer handwashing frequency and own behaviour [44]. Rates may be improved by further enhancing the perception that other children are frequently practising handwashing and thus appealing to the motive of affiliation—a motive rated among the most important by children in our study.

In close alignment to some of the leading frameworks developed to describe the determinants of handwashing, the determinants we have identified in this study span multiple domains. The BCD framework puts forth that three domains of the environment—the physical, social and biological domain—determine behaviour (by acting as stimuli for an individual’s brain, causing a change in their psychology and thereby in their behaviour) [27]. Similarly, the IBM-WASH framework organises determinants into three dimensions—the contextual, psychosocial and technological dimension [45]. The determinants we report here span the physical environment (access to handwashing technology, ease of use, etc.), the biological environment (e.g. environmental dirtiness inducing feelings of disgust) and the social environment (social norms, expectations, perceptions, stigma, etc.), and we find that the humanitarian context influences each of these domains, as well as dictating other factors falling slightly outside of their scope, such as exposure to hygiene promotion—a modification of the community context which is better described under the contextual dimension of the IBM-WASH framework. Recognising the diverse nature of handwashing determinants, humanitarian actors should look to multi-pronged approaches which alter the physical, social and biological environment in ways which both enable and motivate handwashing behaviour.

Our study had various limitations. Though friendship-paired interviews encourage participation, they bring the risk that children may influence each other’s thoughts and responses [46]. In these interviews, we asked children to rate motives using cartoon pictures. We cannot be certain that our depiction of the motives was interpreted as intended; this may be especially true for the more abstract motives such as curiosity, justice and status. However, there are currently no other tools available to measure these motives and we are encouraged by the alignment between emergent themes in the interviews and many of the motive ratings. Future studies may consider using video clips or real-life scenarios to portray motives and should check the understanding of each motive by asking participants to feedback their interpretation of each.

Another limitation related to the motive rating exercise was the qualitative sample size used in this study. Whereas logistic regression was useful in understanding how the different motives were rated among the study participants, it cannot be used to make population-wide inferences because this was a qualitative sample.

We report the perceived determinants of child handwashing. Discrepancies may exist between perceived and actual determinants. There was evidence of this in our study; most respondents believed that knowledge of disease and the benefits of handwashing are strong determinants of children’s handwashing and that increased messaging would improve handwashing rates. However, it was apparent that handwashing promotion exposure was already very high in this camp and an increase is unlikely to impact children’s handwashing rates. A study among adults in refugee camps similarly found that high exposure to hygiene messaging is not associated with high rates of handwashing with soap [37]. Despite this, we are confident that there is some alignment of perceived and actual determinants as we find convergence in the perceived and actual access to handwashing materials.

Finally, our study was limited to one camp, with a largely homogenous population, in one humanitarian context and thus, our findings may not be generalisable beyond this context. The humanitarian sector would benefit from similar studies in different humanitarian camps to determine if children’s handwashing determinants and motivational drivers are common across these contexts, or to what extent handwashing interventions must be tailored to each context.

## Conclusion

This is the first study of handwashing determinants among children in a humanitarian context. Future handwashing interventions for children in humanitarian emergency contexts should go beyond just the provision of basic soap and water facilities and hygiene messaging and give equal consideration to their quality and location. Besides infrastructural improvements, interventions should harness existing social norms and consider using a set of motivational drivers to improve rates of handwashing with soap among children living in humanitarian settings.

## Supporting information

S1 Appendix Interview guides. (PDF)

S2 Appendix Motive pictures and terms. (PDF)

S3 Appendix Coding structure. (PDF)
