# Supplementary material for: Child handwashing in an internally displaced persons camp in Northern Iraq: A qualitative multi-method exploration of motivational drivers and other handwashing determinants
Source: PLoS One. 2020 Feb 3;15(2):e0228482. doi: 10.1371/journal.pone.0228482 (PMC6996827; doi:10.1371/journal.pone.0228482)
Supplement: S1 Appendix — (PDF) [file pone.0228482.s001.pdf]

S1 Appendix: Interview Guides

Guide for Caregiver Semi-Structured Interviews

After obtaining consent and permission to record the interview, proceed with this introduction and the questions below.

My name is Julie Watson I work with the London School of Hygiene. Thank you for meeting with me today.

[The following questions serve as a guide for discussion but can be flexible so long as the topic of conversation remains on the broader context of child hygiene and handwashing]

Introductory questions

1. Tell me about your who lives in the home?

Handwashing practices in the home

We'd like to know a little more about your hygiene in your community and homes.

2. How would you describe the current handwashing practices in the community?
3. Are child handwashing practices different to those of adults in the community?
4. Where do you tend to wash hands? Is this the same for children?
5. What do you think is the best way to wash your hands?
6. Is it important for children to wash their hands? Why?
7. At what times is it important for children to wash their hands?
8. What do children use to wash their hands?
9. Do they wash their hands on their own or are they helped/supervised?
10. During the typical day does anyone in the household remind children to wash their hands?

Soap

11. Where do you get your soap from?
12. How often do you get soap?
13. What do you use soap for?
14. Why is soap used to wash hands? What will happen if it is not used?
15. Do children in your home use soap for handwashing?

Exposure to hygiene promotion

16. How often do you have contact with hygiene promoters?
17. What information do they share about handwashing? Do they talk about child handwashing?
18. Do they directly talk to the children in your household?
19. What have you learnt about child handwashing from the hygiene promoters?
20. To what extent do the messages they give motivate you and your family to wash their hands?

Barriers to handwashing

21. Are there things that prevent the children in your household from washing their hands when they want to, or when you want them to? (*If yes, for each barrier mentioned, ask the likelihood of this occurring and what could be done to remove barrier).* Probe specifically for convenience of location of handwashing materials (soap, water)

Factors that promote handwashing

22. What do you think would help children to practice handwashing more often?
23. What do you think would encourage children to use soap when they wash their hands?

Roles and Responsibilities

24. What role do you think parents take in their children washing their hands?
25. Who else do you think has a role to play in children washing their hands with soap?

Ending question

26. Are there any last comments or questions before we wrap in the discussion?

Guide for Hygiene Promoter Semi-Structured

After obtaining consent and permission to record the interview, proceed with this introduction and the questions below.

My name is Julie Watson; I work with the London School of Hygiene. Thank you for meeting with me today.

[The following questions serve as a guide for discussion but can be flexible so long as the topic of conversation remains on the broader context of child hygiene and handwashing]

Introductory questions

1. Tell me about your experience as a hygiene promoter in Sharia. How did you become involved?
2. What is the best/worst part of your position as a Community Health Promoter?
3. Tell me about your typical day working as a hygiene promoter?

   Probe for:

   a. What time do you start?
   b. How many houses do you visit?
   c. How long do you spend with each house?
   d. What are the things you typically talk about?
   e. Do you talk to other people during the day?
   f. Who do you report to at the end of the day?

   NOTE: If the HP has trouble discussing a TYPICAL day, this can be rephrased as "yesterday" or a specific day in the past. If the HPs activities change day by day, then ask about multiple days – the goal is to get a sense of the HPs typical activities.

Child Hygiene questions

4. Do you think child handwashing is a problem within the community that you serve?
5. What are the current child handwashing practices in the community?
6. Do you talk about child handwashing during your working day?
7. What do you find the general opinion is to child handwashing in the community?

Changing Behavior

8. What are some ways that you have successfully changed hygiene behaviours of children in your community?
9. What type of information and messages do you provide?
10. What are the things that motivate the children you work with to change their practices?
11. How do you tailor your interactions with families?

Barriers to handwashing

12. What do you think gets in the way of children practicing handwashing?
13. What do you think gets in the way for children using soap when they wash their hands?

Ways to promote child handwashing with soap

14. What do you think would encourage children to wash their hands with soap more often?
15. What would help you to promote children handwashing with soap?

Roles and responsibility

16. What role to you think parents have in the children handwashing?
17. Who else do you think has a role in promoting child handwashing with soap?

Ending question

18. Is there any last comments or questions before end the interview?

Guide for Child Paired-Interviews

After obtaining oral consent and permission to record the interview from primary caregivers, and assent from children, proceed with this introduction and the questions/activities below.

Welcome. My name is Julie Watson; I work with the London School of Hygiene. Thank you for meeting with me today. (The following participatory activities should be used to facilitate a discussion around the broader context of hygiene and handwashing and should be used to elicit information according to the topic guide below)

Topic Guide:

- Current handwashing practices
  - When/where do you wash your hands?
  - What do you use to wash your hands?
- Perception of handwashing/perceived importance of handwashing
  - Why do people wash their hands?
  - Do you think it is important?
  - Do you think soap is important?
- Motivations for handwashing
  - How do you feel when you wash your hands?
  - Does anyone help you or tell you to wash your hands?
- Perceived barriers and constraints to practicing handwashing
  - Is there anything stopping you washing your hands when you want to?
  - What about soap? Probe for convenience of location of handwashing materials – soap and water
  - What would make handwashing easier?
- Exposure to handwashing promotion
  - Who talks to you about handwashing?
  - What do they say?
- Important driving motives for children
  - What values and motives drive behavior?

.

Participatory tools to facilitate the conversation:

1. Word associations

This tool is used as an icebreaker, to lead the topic towards hygiene and to understand some of the mental associations children have with handwashing and associated domains.

Methods:

(i) The facilitator will call out the following words and ask the children to say the very first things that comes to mind when they hear it. Words will include:

- Mother
- Father
- Dirt
- Clean hands
- Disgusting

- Water
- Handwashing
- Soap
- Toilet
- Diseases
- Poo
- Sink
- Home
- Smelly
- Fingernails
- Dirty hands
- Clean hands

2. Function of handwashing behaviour

The following method will be used to get an understanding of the function that handwashing with soap serves from the perspective of the child.

Methods:

(i) Children will be asked to shout out all the reasons the can think of why someone would wash their hands. Whilst they are doing this the facilitator will write these down on separate pieces of paper to remind children later of the reasons they have listed. This will continue until the children cannot think of any more reasons.

(ii) The children will then be asked to decide which of these reasons they think are the most important and to explain their choices.

3. Routine scripting

This method will be used to understand the children's daily routine, where handwashing fits in and to identify and barriers to handwashing with soap.

Methods:

(i) The children will be asked to go through everything they typically do in a day from the time they wake up to the time they go to bed.

(ii) The facilitator will arrange images depicting these activities as they speak according to the timeline children give.

(iii) The facilitator will prompt the children to add events they might have missed around using the toilet, bathing, washing their hands, cooking etc.

(iv) The facilitator will ask the children more details about the key moments for handwashing, e.g. why or why did they not hand wash, who was there, where it took place, what was used (e.g. soap, nothing) and why, etc.

4. Pictorial vignettes of critical handwashing junctures

This method aims to elicit social norms around handwashing and perceived barriers to handwashing.

Methods:

(i) Children will be shown various pre-developed pictorial vignettes depicting different handwashing scenarios involving children. The facilitator will explain what is happening

in the vignettes and will ask the children how they think the person in the picture feels when they do these behaviours, how they themselves would feel when they do these behaviours and how others would view them.

Examples of vignettes are:

- A child coming out of a toilet and washing their hands
- A child coming out of the toilet and not washing their hands
- A child playing outside who has fallen over and hands dirty hands and does not wash their hands
- A child who is about to eat and has not washed their hands
- A child washing their hands before helping his/her mother do the cooking

(ii) The facilitator will also use these vignettes to probe the child to talk about how and when they would wash the hands in this scenario if they wanted to and the barriers and enablers to do so.

5. Ideal handwashing facility drawing/description

This method is used to elicit perceived barriers to practice handwashing with soap.

Methods:

(i) The group will be asked about their current handwashing facility, what they like what they do not like about it, what's at the handwashing facility etc.

(ii) On a large piece of paper, placed in the middle of the group, the children will then be asked to draw their ideal handwashing facility, describe where it would be, who would use it, what products would be there, etc.

(iii) The facilitator will prompt them to include detail and will follow on by asking them how this is different to their current handwashing facility and how they would feel using this new handwashing facility.

6. Perceived social norms

This method is used to understand the perceived social norms around handwashing.

Methods:

(i) 10 counters will be given to each child to depict other children in the camp. Children will be asked: 'if we went around the camp today and asked 10 children the questions below, how many children do you think would':

a. Have soap in their houses right now?
b. Would have a place to wash their hands inside their house?
c. Wash their hands with soap after they go to the toilet to have a wee?
d. Wash their hands with soap after they go to the toilet to poo?
e. Wash their hands with soap before they eat?
f. Wash their hands if they got dirt/mud on them?

- Children will also be asked to give explanations for their estimates
