# Supplementary material for: Child handwashing in an internally displaced persons camp in Northern Iraq: A qualitative multi-method exploration of motivational drivers and other handwashing determinants
Source: PLoS One. 2020 Feb 3;15(2):e0228482. doi: 10.1371/journal.pone.0228482 (PMC6996827; doi:10.1371/journal.pone.0228482)
Supplement: S2 Appendix — (PDF) [file pone.0228482.s002.pdf]

S2 Appendix: Motive pictures and terms

STATUS	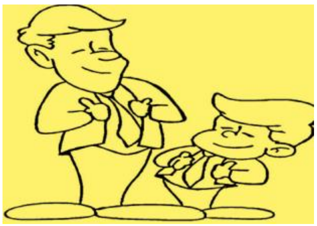	FEAR	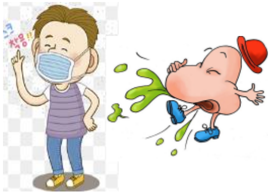
	I want my neighbours to think I am a very important person		I never want to catch germs or diseases
NURTURE	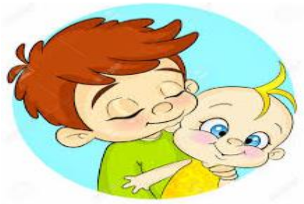	PLAY	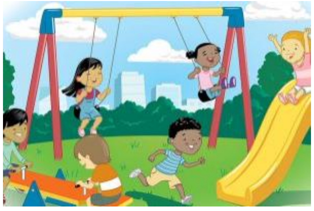
**Term**	I want to take good care of my little brother/sister/cousin		I want to have lots of things to play with
AFFILIATION	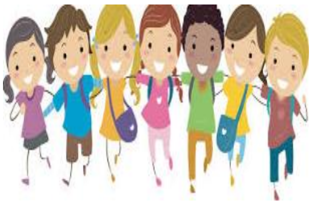	COMFORT	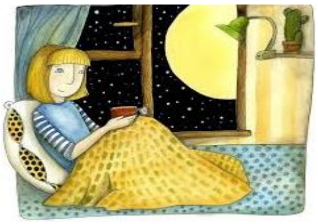
	I always want to have lots of friends		I want my house to be very comfortable
ATTRACT	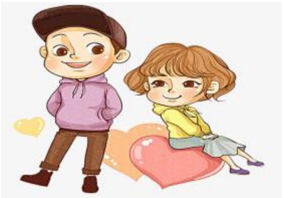	HOARD	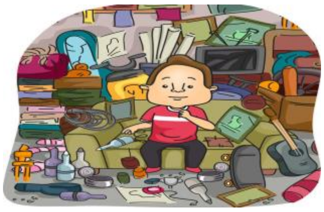
	I always want to look beautiful/handsome		I want to have lots of things in my house
LOVE	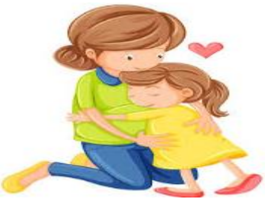	JUSTICE	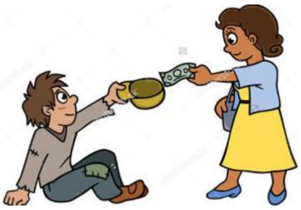
	I always want to feel loved		I want to make people be fair and honest

HUNGER	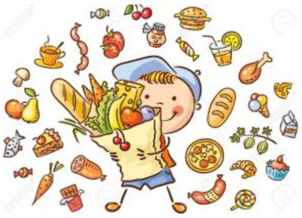	CREATE	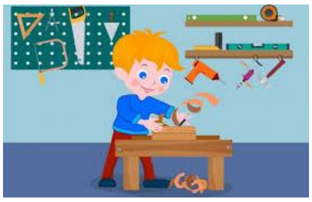
	I always want to have lots of food to eat		I want to be able to make new things
DISGUST	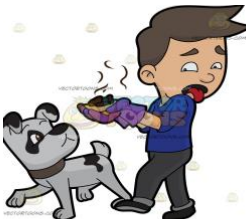	CURIOSITY	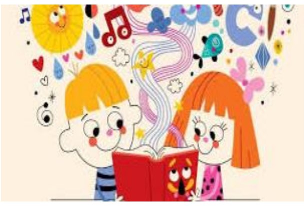
	I never want to be near disgusting, smelly things		I want to learn lots of new things
