# Supplementary material for: Child handwashing in an internally displaced persons camp in Northern Iraq: A qualitative multi-method exploration of motivational drivers and other handwashing determinants
Source: PLoS One. 2020 Feb 3;15(2):e0228482. doi: 10.1371/journal.pone.0228482 (PMC6996827; doi:10.1371/journal.pone.0228482)
Supplement: S3 Appendix — (PDF) [file pone.0228482.s003.pdf]

S3 Appendix: Coding structure

Theme	Associated Codes	Example quote
**FAMILIAL ROLE** *The family's influence and responsibility over children's handwashing behaviour*	Children as role models	*Child: "..little children will learn from us, so if we do not wash our hands they also do not wash their hands too"*
	Children with childcare duties	*Child: "I always care for my little sister and brother and take them outside when I go to play"*
	Parental responsibility	*Child: "everything is available, but it depends on their parents."* *Caregiver: "I think that the parents have a really big role in making the children wash their hands more"* *Hygiene promoter: "I think that the mother has a big role for her children because she spends most of her time with them"*
	Too many children	*Caregiver: "some of my neighbours have many children so it is hard to make them all wash their hands."* *Hygiene Promoter: "I think this carelessness (children not handwashing) comes from the big number of children in one family"*
**ENVIRONMENTAL BARRIERS** *Physical deterrents to children practising handwashing with soap*	Latrines dirty	*Child: "all the latrines are so dirty so we cannot wash our hands"* *Caregiver: "nobody can wash hands in the latrines because they are very dirty"* *Hygiene Promoter: "the toilets are common in this camp. This increases the germs and therefore makes it harder to clean"*
	Communal water points dirty	*Child: "water points which are near our section are so dirty, people throw food into it"* *Caregiver: "because they are so dirty" (when asked why children don't use the water points for handwashing)*
	Lack of soap at latrines	*Child: "…the soap is not available at the toilet so we must go home to wash them"* *Caregiver: "when they go to the toilets, they use only water because there is no soap there"* *Hygiene promoter: "no, I went there but I could not find it (soap at the latrines)"*
	Communal soap dirty	*Child: "..even if there is soap, I do not think they would use the same soap for everyone because I think it is a dirty thing"*
	Lack of water at latrines	*Child: "yes there is no soap in the latrines even sometimes there is no water"* *Caregiver: "the problem is that all the latrines are dirty and so far from our house, and sometimes the water is not available there so the children can't wash their hands"*
	Cold water	*Child: "we cannot use latrines for washing hands because there is no soap and warm water, just cold water and they are very dirty"*

		<u>Caregiver</u>: *"i*f the latrines were closer for the children and if they had warm water, they would wash their hands more for sure"
	Latrine block distance	<u>Child</u>: *"if they were far from the latrines, they would not wash their hands, if they were near to latrines, they would wash their hands."*<br><u>Caregiver</u>: *"if the latrines were closer for the children and if they had warm water, they would wash their hands more for sure"*
	Lack of electricity	<u>Child</u>: *"some nights there is no electricity or water at latrines, we are frightened to go to wash our hands, so it is hard for us to wash our hands"*<br><u>Caregiver</u>: *"the latrines are far away from my children and also the electricity in not always available for us and the water is also not always available."*
	Shared facilities	<u>Child</u>: *"I wish every household had its own toilet to keep our toilet clean and so we could use our own soap after using the toilet …"*<br><u>Caregiver</u>: *"there wouldn't be a problem if each household had its own bathroom and toilet so the children could wash their hands easily in the sink and with soap"*<br><u>Hygiene Promoter</u>: *"the toilets are common in this camp. This increases the germs and therefore makes it harder to clean ..."*
**HYGIENE PROMOTION EXPOSURE**<br>*Perception of hygiene promotion and existing exposure*	Seven steps of handwashing	<u>Child</u>: *"we use soap for handwashing, we wash our hands after using the toilet and before eating according to the seven steps"*<br><u>Caregiver</u>: *"they (hygiene promoters) tell me that when our children go to the toilets, they should wash their hands with the seven steps"*<br><u>Hygiene promoter</u>: *"I see that the seven steps of handwashing is a really successful way to show the children how to wash their hands"*
	Give them awareness	<u>Child</u>: *"if we tell them all about handwashing, how handwashing is important and if you wash your hands you will be healthy, they will wash their hands."*<br><u>Caregiver</u>: *"if the hygiene promoters, CFS and school tell them about handwashing they will wash their hands more because they like it and they will listen to you more than us"*<br><u>Hygiene promoter</u>: *"the school should increase its education in the field of health awareness and awareness in the home is very low - we should increase this percentage"*
	Past hygiene promotion exposure	<u>Child</u>: *"some NGOs come to our home and give an awareness seminar about handwashing ..."*

		<u>Caregiver</u>: *"they hear these messages all around the camp, for example at school, CFS and some other places"*<br><u>Hygiene promoter</u>: *"we go to kindergartens, schools and homes to provide health awareness to them and we focus on the methods of washing hands a lot because it is the basis of human cleanliness"*
	Increase in children's handwashing after hygiene promotion	<u>Hygiene promoter</u>: *"The percentage of handwashing in the camp is not 100%, but the percentage has increased after the entry of the organization and we have increased this through the dissemination of awareness.."*
	Out-of-school children practising less handwashing	<u>Hygiene Promoter</u>: *"the reason for the little washing of the hands is that some children do not go to schools and kindergartens"*
	Knowledge of disease transmission	<u>Child:</u> *"we should wash our hands well because dirtiness goes under nails, so when we eat food, germs go into our body and we will be sick"*
	Knowledge of specific diseases	<u>Child</u>: *"Handwashing is so important for us to avoid cholera"*<br><u>Caregiver</u>: *"… in these last few months mumps is so common in the camp, my little girl had mumps"*<br><u>Hygiene promoter</u>: *"there are not many diseases, but some are widespread, such as cholera, lice, scabies and diarrhoea"*
	Handwashing to avoid diseases	<u>Child:</u> *"handwashing is so important for us to avoid cholera. We should wash our hands using soap. If we do not wash our hands, we will be sick"*<br><u>Caregiver</u>: *"I always tell my children to wash their hands to avoid diseases and to look clean"*<br><u>Hygiene promoter</u>: *I tell them not to let their children play near the dirt and explain the diseases that affect humans because of the dirt and that the parents should continue to follow their children's handwashing"*
	Hygiene promotion popular with children	*Caregiver: "they enjoyed hearing messages because the hygiene promoters were showing them some posters"*
	Hygiene promotion decreasing	*Caregiver: "in the beginning when we came to the camp, they (hygiene promoters) were coming a lot, but now they are not coming as often"*
**PRESCRIPTIVE SOCIAL NORMS**<br>*Rules specifying handwashing behaviour that persons ought to engage in*	Avoiding dirty people	<u>Child</u>: *"If my hands smell good, people will not try to avoid me, and I will have lots of friends"*
	Social stigma	<u>Child:</u> *"we want our hands to be clean and look to nice, so other children do not laugh at us"*
	Cleanliness a reflection of character	<u>Child</u>: *"they say that he is a polite, good boy and is clean (if he washes his hands)"*

	Cleanliness a reflection of the family	<u>Child</u>: *"they will say he is from a clean family and they are good that they wash their hands"*
	Handwashing to look nice for others	<u>Child</u>: *"children wash their hands to look nice. If they go somewhere and their hands are dirty people will blame them"*
	Handwashing to smell nice for others	<u>Child</u>: *"I wash my hands because I want to smell nice before going to the school"*
	Making parents proud	<u>Child</u>: *"our parents are proud of us when we wash our hands, they always encourage us to wash our hands"*
